# Sericin Supplementation Prior to and During In Vitro Maturation Mitigates H_2_O_2_‐Induced Oxidative Stress in Bovine Oocytes

**DOI:** 10.1155/vmi/6119294

**Published:** 2026-05-21

**Authors:** Sirawit Yindeetrakul, Supawit Triwutanon, Anawat Sangmalee, Theera Rukkwamsuk

**Affiliations:** ^1^ Department of Large Animal and Wildlife Clinical Sciences, Faculty of Veterinary Medicine, Kasetsart University, Kamphaeng Saen Campus Kamphaeng Saen, Nakhon Pathom, 73140, Thailand, ku.ac.th

**Keywords:** bovine oocyte, in vitro maturation, oxidative stress, sericin

## Abstract

Oxidative stress during oocyte handling and in vitro maturation (IVM) can impair meiotic competence and compromise developmental potential in bovine oocytes. Thus, this study aimed to evaluate whether sericin supplementation during both prior to IVM and IVM phases could mitigate hydrogen peroxide (H_2_O_2_)–induced oxidative stress. All oocytes were cultured in tissue culture medium (TCM‐199) at 38.5°C under 5% CO_2_ and allocated into three groups: (1) the control group, without H_2_O_2_ induction and without sericin, (2) the oxidative stress group, exposed to 100‐μM H_2_O_2_ for 1 h in collecting media and subsequently cultured in standard medium (−SC), and (3) the sericin‐supplemented group, exposed to H_2_O_2_ under the same condition as −SC group but supplemented with 0.1% sericin in the collecting medium and 0.05% sericin in the maturation medium (+SC). After culture, two parameters were assessed: nuclear maturation to the metaphase II (MII) stage, evaluated by aceto‐orcein staining, and malondialdehyde (MDA) levels, as an indicator of lipid peroxidation. The maturation rates were 70.36 ± 3.94%, 32.62 ± 5.89%, and 74.56 ± 6.05% for the control, −SC, and +SC groups, respectively, with significant differences observed between the −SC group and the control and +SC groups (*p* < 0.001). The corresponding MDA levels were 1.97 ± 0.14, 2.94 ± 0.28, and 1.09 ± 0.32 μM for the control, SC−, and SC + groups, respectively. The + SC group showed significantly lower MDA levels than both the control (*p* < 0.05) and the −SC groups (*p* < 0.001). These findings demonstrated that sericin supplementation attenuates oxidative stress–associated lipid peroxidation and preserves nuclear maturation in bovine oocytes under experimentally induced oxidative stress conditions, supporting its potential use as an antioxidant supplement during oocyte handling prior to IVM.

## 1. Introduction

Reproductive technologies play a key role in enhancing both productivity and genetic improvement, contributing to long‐term economic sustainability and the conservation of indigenous breeds. Among these technologies, in vitro embryo production (IVEP) has become particularly valuable, consisting of sequential procedures beginning with oocyte retrieval and followed by in vitro maturation (IVM), fertilization (IVF), and culture (IVC) to generate embryos suitable for transfer or cryopreservation [1]. The developmental potential of oocytes is influenced by numerous intrinsic and extrinsic factors, including donor physiology, nutritional status, and environmental conditions such as ambient temperature and photoperiod, which can modulate follicular dynamics and oocyte competence [2]. In addition to these factors, oxidative stress represents a major threat to oocyte quality. Excessive reactive oxygen species (ROS) can disrupt maturation and compromise early embryogenesis during both preculture handling and subsequent IVC phases [3, 4]. These ROS induce oxidative damage through protein and DNA oxidation, lipid peroxidation, mitochondrial dysfunction, and developmental arrest [5].

Under physiological conditions, oocytes develop within a finely regulated follicular microenvironment in which ROS and antioxidant defenses maintain a delicate redox balance [6]. Disruption of this balance, such as during ovary collection, transportation, temperature fluctuation [7, 8], or postmortem ischemia, can lead to excessive ROS accumulation before IVM, leaving oocytes highly vulnerable at an early stage [9]. Oocytes retrieved for IVM may already possess compromised antioxidant capacity due to intrinsic follicular factors, including mitochondrial activity, metabolic demands, and the limited stores of enzymatic and nonenzymatic antioxidants in both the oocyte and its surrounding cumulus cells [6]. A study similarly highlighted that the laboratory environment in assisted reproductive technologies inherently predisposes gametes to ROS overproduction, often even before oocyte maturation begins [10]. Collectively, these findings indicate that the period prior to IVM represents a particularly sensitive window during which unmanaged oxidative stress can impair meiotic progression, cytoskeletal integrity, and subsequent developmental competence. ROS production during IVM exhibits dynamic fluctuations, with increases occurring during the maturation process in association with metabolic and redox changes within oocytes [11]. This increase coincides with the oocyte’s heightened adenosine triphosphate (ATP) demand during critical processes, such as spindle formation, chromosome segregation, and cell division [12]. In cellular systems, excessive ROS initiates lipid peroxidation, leading to the formation of reactive aldehydes such as malondialdehyde (MDA). MDA, a stable end product of lipid peroxidation, is widely used as a biochemical marker of oxidative stress across mammalian cell systems [13].

To mitigate oxidative damage, the use of biomolecules with antioxidant properties has gained significant interest for improving oocyte quality. Sericin, a silk‐derived protein from silkworm (*Bombyx mori*) [14], has attracted attention due to its high biocompatibility and strong antioxidant capacity [15, 16]. Supplementation of culture media with sericin has shown to stimulate collagen production and promote cell proliferation and adhesion [17]. Growing evidence further supports its application in reproductive systems; supplementing maturation media with 0.05% sericin enhances meiotic progression, reduces intracellular hydrogen peroxide (H_2_O_2_) accumulation, and decreases DNA fragmentation in oocytes exposed to oxidative stress [18]. In addition, it was reported that supplementation with 0.1% sericin in both collection and maturation media improved oocyte maturation [19]. In embryos, sericin has also been shown to promote preimplantation development and reduce ROS‐induced DNA damage, particularly under experimentally induced oxidative stress, as demonstrated in bovine embryos cultured individually [20].

Limited information is available regarding the protective role of sericin, particularly during the pre‐IVM phase, a critical window when oocytes are highly vulnerable to oxidative insult following follicular removal. Moreover, the effects of sericin supplementation during both pre‐IVM and IVM phases under a defined oxidative stress model have not been fully elucidated. A recent study demonstrated that short‐term exposure to 100‐μM H_2_O_2_ for 1 h provides a reliable in vitro model for mimicking acute oxidative insults encountered during prior to IVM handling of bovine oocytes, offering a standardized platform for evaluating antioxidant interventions [21]. While that study focused on optimizing and validating an oxidative stress model prior to IVM, it did not evaluate antioxidant interventions or functional outcomes of oocyte quality. Building upon this model, the present study aimed to investigate the protective effects of sericin supplementation during both the pre‐IVM and IVM phases by assessing nuclear maturation and lipid peroxidation in bovine oocytes. Unlike earlier studies that primarily explored sericin as a serum substitute, this study focuses on its role as an active antioxidant supplement in conventional culture media. The sericin concentration used during IVM was selected based on previous findings demonstrating that this dosage optimizes meiotic progression and reduces intracellular ROS levels without causing cytotoxic effects [18–20], making it an appropriate and physiologically safe concentration for improving oocyte competence under oxidative stress conditions.

## 2. Materials and Methods

### 2.1. Ovary Collection and Handling

The oocytes used in this study were collected from ovaries of healthy mature female cattle slaughtered at three slaughterhouses in Nakhon Pathom Province. Oocyte collection was conducted between June and September 2025 to minimize the potential effects of seasonal variation and heat stress on oocyte quality. Bovine ovaries were transported to the laboratory within 2 h of collection and maintained at 30°C–35°C in 0.9% NaCl supplemented with 100 IU/mL penicillin and 100 μg/mL streptomycin sulfate (HyClone, Logan, UT, USA) during transport to minimize microbial contamination.

### 2.2. Cumulus–Oocyte Complex (COC) Recovery and Selection

Upon arrival at the laboratory, the ovaries were washed three times with prewarmed (37°C) transport medium. COCs were then aspirated from follicles measuring 2–5 mm in diameter using an 18‐gauge needle attached to a 10‐mL disposable syringe. Only Grade 1 and Grade 2 COCs, characterized according to the morphological criteria established by the International Embryo Technology Society (IETS), were selected for further processing.

### 2.3. Induction of Oxidative Stress and Grouping

Before IVM, COCs were randomly assigned into three experimental groups: control, –SC (without sericin supplementation), and +SC (sericin‐supplemented). Each treatment group was conducted in five independent replicates, using oocytes collected from different ovaries for each replicate. In each replicate, approximately 25–30 oocytes per group were subjected to IVM. All groups were transferred using a mouth pipette into a collecting medium consisting of phosphate‐buffered saline (PBS; pH 7.4; Fisher BioReagents, Thermo Fisher Scientific) supplemented with 10% fetal bovine serum (FBS; HyClone, Cytiva, Marlborough, MA, USA), 100 IU/mL penicillin, and 100 μg/mL streptomycin (HyClone, Cytiva, USA).

For the –SC and +SC groups, H_2_O_2_ (H_2_O_2_; 30% w/w analytical grade, ACS reagent, Fisher Scientific, USA) was freshly added to the collecting medium to achieve a final concentration of 100 μM, and the COCs were incubated at 38.5°C for 1 h to induce oxidative stress. This concentration was selected based on our previous optimization study, which demonstrated that exposure to 100‐μM H_2_O_2_ reliably induces oxidative stress in bovine oocytes while preserving oocyte viability and avoiding acute cytotoxic effects [21]. The + SC group additionally received 0.1% (w/v) sericin (Wako Pure Chemical Industries, Osaka, Japan) in the collecting medium. This higher concentration was selected based on previous reports demonstrating its effectiveness during oocyte handling and was applied during the pre‐IVM phase to counteract acute oxidative insult occurring immediately after follicular removal. The control group was handled identically but without H_2_O_2_ or sericin treatment.

### 2.4. IVM

After oxidative stress induction, all COCs were washed three times and transferred to maturation medium. The +SC group continued to receive 0.05% (w/v) sericin supplementation during IVM, while the control and –SC groups were cultured in maturation medium without sericin. A lower concentration (0.05%) was used during IVM based on previous studies reporting significant improvement in meiotic progression within the range of 0.05%–0.1% and was selected as the minimal effective dose to reduce potential interference with physiological redox signaling and to enhance practical applicability in routine laboratory conditions. Selected COCs were placed into 50‐μL drops of maturation medium at a density of 10 oocytes per drop. The oocytes were then cultured at 38.5°C under a humidified atmosphere of 5% CO_2_ in air for 23 h. The maturation medium was based on Medium‐199 (M‐199; Cytiva, USA) supplemented with 10 IU/mL equine chorionic gonadotropin (Folligon; Intervet, Boxmeer, Netherlands), 10 IU/mL human chorionic gonadotropin (Chorulon; Intervet, Netherlands), 100 IU/mL penicillin, and 100 μg/mL streptomycin sulfate. For the sericin‐supplemented group, 0.05% (w/v) sericin was added in the medium. All maturation media were preequilibrated for 2 h in a humidified CO_2_ incubator prior to use.

### 2.5. Aceto‐Orcein Staining

Following incubation, cumulus cells were removed by repeated pipetting in PBS containing 0.25% hyaluronidase (MedChemExpress, Monmouth Junction, NJ, USA). The denuded oocytes were then fixed in methanol:acetic acid (3:1 v/v) for 48 h. After fixation, oocytes were stained with 2% aceto‐orcein (prepared in 45% acetic acid) for 10 min. The nuclear maturation stage of each oocyte was subsequently assessed under a light microscope (Olympus CX22LED, Olympus, Tokyo, Japan) at 400× total magnification [19].

### 2.6. Thiobarbituric Acid Reactive Substances (TBARSs)

Intracellular MDA was quantified using a TBARS assay adapted and modified from previously described methods [22–24]. For each experimental group, 100 of matured oocytes were denuded of cumulus cells and processed together as a single biological sample, with the procedure repeated in three independent replicates. The denuded oocytes were suspended in 30 μL of PBS and homogenized by brief sonication to obtain a uniform oocyte homogenate. A 30‐μL homogenate was mixed with 50 μL of glacial acetic acid and incubated at 37°C for 1 h. The samples were then centrifuged at 5000 × g for 10 min. A 60‐μL aliquot of the resulting supernatant was combined with 60 μL of 3.5 M sodium acetate buffer and 60 μL of 0.8% thiobarbituric acid (Sigma‐Aldrich, St. Louis, MO, USA). The mixture was heated at 95°C for 1 h, followed by incubation on ice for 30 min. Samples were then centrifuged at 1500 × g for 10 min at 4°C. The resulting supernatant was measured for absorbance at 532 nm using a microplate reader (Sunrise Absorbance Microplate Reader, Tecan Group Ltd., Männedorf, Switzerland) and compared against a standard curve generated using 1,1,3,3‐tetraethoxypropane (Sigma‐Aldrich, USA).

### 2.7. Data Analysis

The normality of data distribution was assessed using both Shapiro–Wilk and Kolmogorov–Smirnov tests. All variables met the assumption of normality (*p* > 0.05). Homogeneity of variance was verified using Levene’s test before conducting further analysis. Comparisons among treatment group means were performed using one‐way ANOVA, and multiple comparisons between groups were evaluated using Tukey’s honestly significant difference (HSD) test.

## 3. Results

### 3.1. Oocyte Maturation Assessment

**FIGURE 1:**
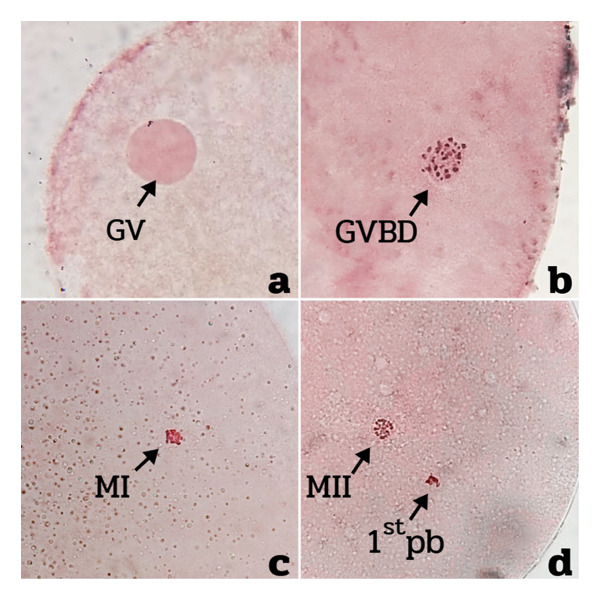
Representative images of bovine oocytes at different nuclear maturation stages following aceto‐orcein staining: (a) germinal vesicle (GV), (b) germinal vesicle breakdown (GVBD), (c) metaphase I (MI), and (d) metaphase II (MII) with first polar body (1^st^ pb) extruded.

Bovine oocytes at different nuclear maturation stages are shown in Figure 1. The results showed that the oxidative stress group (–SC) had a significantly higher proportion of oocytes arrested at the germinal vesicle (GV) and germinal vesicle breakdown (GVBD) stages, as compared with both the control and sericin‐supplemented (+SC) groups (*p* < 0.01), indicating meiotic arrest under oxidative stress conditions. Conversely, the percentage of oocytes reaching the metaphase II (MII) stage was markedly reduced in the –SC group compared with both the control and +SC groups (*p* < 0.001). No significant difference in the MII rate was observed between the control and +SC groups (Table 1), suggesting that sericin supplementation effectively restored meiotic progression impaired by oxidative stress. These findings indicated that oxidative stress induced by 100‐μM H_2_O_2_ prior to IVM significantly inhibited meiotic maturation, while sericin supplementation during both the pre‐IVM and IVM phases mitigated oxidative damage and supported nuclear maturation to levels comparable with the control.

**TABLE 1:** Percentage of bovine oocytes at different meiotic stages (GV, GVBD, MI, and MII) after 23 h of in vitro maturation under three treatment groups.

H_2_O_2_ exposed	Medium		Number of oocytes examined	Percentage of oocyte at each stage			
	Collection	IVM		GV	GVBD	MI	MII
0 µM	0% SC	0% SC	118	0^a^	11.77 ± 6.75^a^	17.86 ± 7.71^a^	70.36 ± 3.94^a^
100 µM	0% SC	0% SC	127	21.59 ± 5.21^b^	25.76 ± 9.71^b^	20.02 ± 6.46^a^	32.62 ± 5.89^b^
100 µM	0.1% SC	0.05% SC	122	4.10 ± 0.10^a^	7.40 ± 1.90^a^	13.93 ± 4.57^a^	74.56 ± 6.05^a^
			*p* value	< 0.001	< 0.05	> 0.05	< 0.001

*Note:* Results are presented as mean ± SD, with different superscripts within the same column indicating significant differences (*p* < 0.05).

Abbreviations: GV = germinal vesicle, GVBD = germinal vesicle breakdown, MI = metaphase I, MII = metaphase II, and SC = sericin.

### 3.2. Measurement of TBARS Levels

**FIGURE 2:**
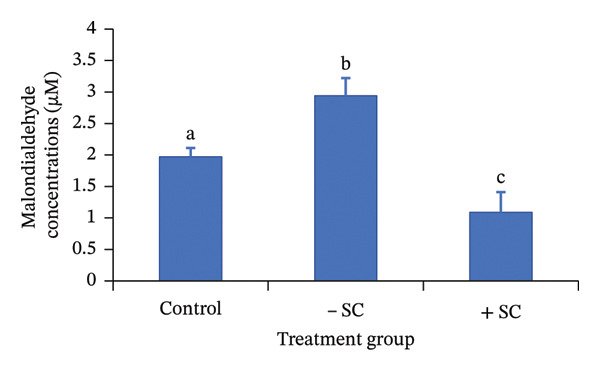
Intracellular malondialdehyde (MDA) concentrations (μM) in bovine oocytes following pre‐IVM oxidative stress induction and subsequent in vitro maturation under control, oxidative stress (–SC), and sericin‐supplemented (+SC) conditions. Values are presented as means, and the error bars are SD. Different letters represented the means significantly differed at *p* < 0.05.

The intracellular MDA levels are presented in Figure 2. MDA levels were measured using the TBARS assay, as previously described, with three replicates performed for each group. The results showed that the H_2_O_2_‐treated group exhibited the highest MDA concentration (2.94 ± 0.28 μM), indicating a pronounced increase in lipid peroxidation compared with the control group (1.97 ± 0.14 μM). In contrast, the sericin‐supplemented group showed the lowest MDA level (1.09 ± 0.32 μM). Statistical analysis revealed that MDA concentrations differed significantly among the three groups (*p* < 0.05).

## 4. Discussion

The oxidative stress induced by H_2_O_2_ before IVM markedly reduced oocyte meiotic competence, as shown by the decreased proportion of oocytes reaching the MII stage. Sericin supplementation restored nuclear maturation to levels comparable to the control, supporting its protective role against oxidative stress and its capacity to promote meiotic progression. Overall, these findings indicated that sericin supplementation effectively counteracts pre‐IVM oxidative stress, modeling oxidative stress conditions relevant to oocyte handling following ovary collection.

The protective mechanism of sericin is thought to be related to its unique antioxidant properties. Sericin, a natural protein derived from the silkworm (*Bombyx mori*), is rich in polar amino acids such as serine, aspartic acid, and glycine. These amino acids provide functional groups (–OH, –COOH, and –NH_2_) capable of donating electrons or scavenging free radicals, thereby reducing oxidative reactions [15, 25]. This activity helps inhibit lipid peroxidation and minimize Fenton reactions that elevate intracellular ROS levels. Additionally, sericin exhibits cryoprotective properties, stabilizing proteins and cell membranes while maintaining moisture balance in culture systems [26]. Collectively, these characteristics may contribute to protection against oxidative damage during meiosis and support proper nuclear maturation. These proposed mechanisms are consistent with the findings of the present study in which the sericin‐supplemented group exhibited the highest proportion of oocytes reaching the MII stage and the lowest MDA levels. To fully elucidate the precise mode of action of sericin, future studies should quantify H_2_O_2_ dynamics in both the extracellular medium and intracellular compartments.

A notable observation in this study was the substantial increase in MDA levels in the –SC group compared with both the control and +SC groups. This elevation indicated that exposure to H_2_O_2_ during the pre‐IVM phase effectively induced oxidative stress, leading to enhanced lipid peroxidation and a corresponding decline in nuclear maturation. Interestingly, oocytes in the control group also exhibited measurable levels of MDA, suggesting that in vitro conditions may inherently expose oocytes to a certain degree of oxidative stress [6, 7]. This aligns with a previous report demonstrating that oocytes can generate ROS during metabolic activation after collection and throughout the early IVM period [11]. The significant reduction in MDA observed in the +SC group not only relative to –SC but also compared with the control indicates that sericin supplementation effectively attenuated lipid peroxidation under oxidative stress conditions and was associated with lower intracellular oxidative damage than that observed under standard IVC conditions. These findings are consistent with previous studies reporting that sericin supplementation improves oocyte maturation and reduces oxidative damage under in vitro conditions [18–20, 27, 28].

Recent evidence also indicated that sericin’s antioxidative action extends beyond direct free radical scavenging to include the preservation of cumulus–oocyte communication. Supplementation with 0.1% sericin has been shown to reduce intracellular ROS, maintain gap junction communication (GJC), and preserve transzonal projections (TZPs) between the oocyte and cumulus cells. Such sustained communication enhances the transfer of metabolites and promotes glutathione (GSH) synthesis within the oocyte, an intrinsic antioxidant crucial for meiotic progression [28]. These dual mechanisms, direct suppression of ROS and indirect enhancement of endogenous GSH, provide a plausible explanation for the superior nuclear maturation seen in the +SC group. The concomitant decrease in MDA levels and increase in MII rate observed in this study thus suggest an improved oxidative environment that supports meiotic resumption, spindle stability, and chromosomal alignment, supporting meiotic progression under oxidative challenge.

Although sericin supplementation demonstrated clear benefits in reducing oxidative damage and improving nuclear maturation, several limitations of this study should be noted. The present study focused primarily on nuclear maturation and lipid peroxidation, without assessing cytoplasmic maturation or intracellular structures that are highly sensitive to oxidative stress [29]. Previous evidence indicated that oxidative stress can impair mitochondrial ATP production, disrupt microtubule polymerization, and compromise spindle assembly, leading to chromosome misalignment and reduced developmental competence [30]. Such cytoskeletal abnormalities have also been associated with decreased fertilization potential and lower blastocyst formation [31]. In this context, oocytes may successfully resume meiosis and undergo early cleavage, yet fail to sustain later embryonic development if cytoplasmic maturation, mitochondrial competence, or spindle integrity is incompletely protected.

In the present study, MDA levels were measured only after completion of IVM to reflect the cumulative oxidative status associated with meiotic progression. Because sericin was supplemented during both the pre‐IVM and IVM phases, the experimental design does not allow discrimination of the relative contribution of each phase. Therefore, the protective effect observed in the +SC group should be interpreted as the outcome of sequential phase‐specific supplementation rather than a single‐stage mechanism. Future studies employing phase‐restricted supplementation or intermediate time‐point analyses would help clarify the temporal role of sericin during oxidative challenge. Additionally, although H_2_O_2_ exposure significantly reduced meiotic competence, a proportion of oocytes in the –SC group still progressed to advanced meiotic stages, indicating the presence of intrinsic endogenous antioxidant defense systems within bovine oocytes and cumulus cells. These endogenous mechanisms may partially buffer oxidative stress but appear insufficient to fully preserve optimal meiotic competence under acute oxidative conditions.

Moreover, because sericin was supplemented simultaneously with H_2_O_2_, the present experimental design does not allow a clear distinction between direct H_2_O_2_ neutralization in the culture medium and downstream suppression of intracellular lipid peroxidation. Therefore, future investigations should include assessments of mitochondrial activity, spindle morphology, and specific oxidative biomarkers, along with downstream functional outcomes such as IVF success and embryo development. These approaches would help clarify whether sericin prevents less obvious cytoskeletal defects that may not be captured by nuclear maturation alone.

## 5. Conclusions

This study demonstrated that sericin supplementation during both prior to IVM and IVM phases effectively mitigated oxidative stress induced by H_2_O_2_ in bovine oocytes. Sericin reduced intracellular MDA levels, a widely accepted indicator of lipid peroxidation, and restored meiotic progression to the MII stage to levels comparable with the control group under oxidative stress condition. These findings suggest a protective role of sericin against oxidative damage during in vitro oocyte handling. However, the present results should be considered preliminary, as the study focused primarily on nuclear maturation and oxidative biomarkers. Further investigations evaluating cytoplasmic maturation, mitochondrial function, spindle integrity, and subsequent fertilization and embryo development outcomes are required to determine the broader biological relevance of sericin supplementation. Validation under IVF conditions and in oocytes derived from ovum pick‐up (OPU) systems will be essential before practical application in routine reproductive biotechnology can be recommended.

## Author Contributions

Conceptualization, S.Y., S.T., A.S., and T.R.; methodology, S.Y., S.T., A.S., and T.R.; software, S.Y.; validation, S.Y. and T.R.; formal analysis, S.Y.; investigation, S.Y., S.T., and A.S.; resources, T.R.; data curation, S.Y.; writing–original draft, S.Y.; writing–review and editing, T.R.; visualization, S.Y. and T.R.; supervision, T.R.; project administration, S.Y.; and funding acquisition, T.R.

## Funding

This research and the APC were funded by the Faculty of Veterinary Medicine, Kasetsart University.

## Disclosure

All authors have read and agreed to the published version of the manuscript. An earlier version of this manuscript was posted as a preprint on Preprints.org [32].

## Ethics Statement

Ethical review and approval were waived for the study because the oocyte samples were obtained from slaughterhouses as part of the routine meat inspection process. The animals were slaughtered for commercial purposes and not specifically for research. Only carcass tissues from animals certified as fit for human consumption by veterinary authorities were used. Therefore, no live animals were handled, subjected to experimental procedures, or exposed to any additional harm or distress for the purpose of this study.

## Conflicts of Interest

The authors declare no conflicts of interest.

## Data Availability

The original contributions presented in this study are included in the article. Further inquiries can be directed to the corresponding author.
